# Alcohol consumption, blood alcohol concentration level and guideline compliance in hospital referred patients with minimal, mild and moderate head injuries

**DOI:** 10.1186/1757-7241-19-25

**Published:** 2011-04-17

**Authors:** Marianne Efskind Harr, Ben Heskestad, Tor Ingebrigtsen, Bertil Romner, Pål Rønning, Eirik Helseth

**Affiliations:** 1 Department of Neurosurgery, Oslo University Hospital - Ullevål, P.O. Box 4950 Nydalen, N-0424 Oslo, Norway; 2 Faculty of Medicine, University of Oslo, P.O. Box 1018 Blindern, N-0315 Oslo, Norway; 3 Department of Neurosurgery, Stavanger University Hospital, P.O. Box 8100, N-4068 Stavanger, Norway; 4 Department of Neurosurgery, Faculty of Health Sciences, Institute for Clinical Medicine, University of Tromsø, and University Hospital of North Norway, P.O. Box 6060, N-9038 Tromsø, Norway; 5 Department of Neurosurgery, Rigshospitalet, Blegdamsvej 9, DK-2100 Copenhagen Ø, Denmark

## Abstract

**Background:**

In 2000 the Scandinavian Neurotrauma Committee published guidelines for safe and cost-effective management of minimal, mild and moderate head injured patients.

The aims of this study were to investigate to what extent the head injury population is under the influence of alcohol, and to evaluate whether the physicians' compliance to the guidelines is affected when patients are influenced by alcohol.

**Methods:**

This study included adult patients (≥15 years) referred to a Norwegian University Hospital with minimal, mild and moderate head injuries classified according to the Head Injury Severity Scale (HISS). Information on alcohol consumption was recorded, and in most of these patients blood alcohol concentration (BAC) was measured. Compliance with the abovementioned guidelines was registered.

**Results:**

The study includes 860 patients. 35.8% of the patients had consumed alcohol, and 92.1% of these patients had a BAC ≥ 1.00‰. Young age, male gender, trauma occurring during the weekends, mild and moderate head injuries were independent factors significantly associated with being under the influence of alcohol. Guideline compliance was 60.5%, and over-triage was the main violation. The guideline compliance showed no significant correlation to alcohol consumption or to BAC-level.

**Conclusions:**

This study confirms that alcohol consumption is common among patients with head injuries. The physicians' guideline compliance was not affected by the patients' alcohol consumption, and alcohol influence could therefore not explain the low guideline compliance.

## Background

Traumatic brain injury (TBI) is the most common cause of death and permanent disability in trauma patients [1-3]. The incidence of TBI varies between different countries and studies [4]. The incidence for hospital referred and fatal TBIs in Europe and the United States is reported to be in the range of 83.3 - 403/100 000 and 15-18/100 000, respectively [3,5-8]. Various studies have shown that 16 - 51% of the patients admitted for TBIs are under the influence of alcohol [9-12].

In year 2000 the Scandinavian Neurotrauma Committee (SNC) published guidelines with regards to safe and cost-effective management of minimal, mild and moderate head injured patients classified according to the Head Injury Severity Scale (HISS) [13,14]. Compliance to these guidelines has been far from complete [15,16]. Over-triage with either unnecessary hospital admission and/or CT-scanning was the main violation resulting in a cost increase for the health care provider. A possible explanation for the low guideline compliance with a substantial over-triage could be the frequent alcohol influence in this patient population. However, the relationship between alcohol consumption and compliance to these guidelines has so far not been studied.

The purpose of this study has been to investigate to what extent patients with minimal, mild and moderate head injuries are under influence of alcohol, and if alcohol consumption affects how these patients are managed according to the SNC guidelines.

## Methods

This study is based on data collected from Stavanger University Hospital, a hospital located in the southwestern part of Norway with a catchment population of 320 000. A search was made in the hospital's electronic medical charts for patients registered with ICD-10 codes involving head injuries referred to the hospital from January through June in the years 2005, 2007 and 2009. Adult patients (≥ 15 years) with minimal, mild or moderate head injuries according to the Head Injury Severity Scale, HISS, were included in the study [14]. The medical record for each patient was retrospectively reviewed and patient information registered in a database.

The recorded patient information included:

a. date of birth, trauma date and gender

b. Glasgow Coma Scale (GCS) score on admission [17]

c. amnesia (yes or no)

d. HISS score (minimal, mild, moderate) [14]

e. hospitalization for overnight observation (yes or no)

f. risk factors present according to SNC guidelines (yes or no) [13]

g. head CT performed (yes or no)

h. related pathological findings on head CT (yes or no)

i. hospitalization required for reasons other than the actual head injury (yes or no)

j. compliance according to SNC guidelines (yes, no - over-triage with unnecessary CT scan, no - over-triage with unnecessary admission for overnight observation, no - over-triage with both unnecessary CT scan and admission or no - under-triage with CT scan not taken and/or not admitted for overnight observation)

k. weekday of admission

l. alcohol consumption (yes (self-reported, patients clinically judged to be under the influence of alcohol by the admitting physician or blood alcohol concentration > 0) or no)

m. in the majority of patients who were judged to be under influence of alcohol based on clinical evaluation and/or reported that they had consumed alcohol, the blood alcohol concentration (BAC) in promille (grams of alcohol per kilogram of blood) was measured on admission.

For statistical analysis variables were checked for normality graphically using quantile-quantile plots and analytically using the Shapiro-wilks test. We used a combination of robust independent t-samples tests, chi squared tests and Wilcoxon tests to check if groups where equal. Univariate and multivariate logistic regression was used after dichotomizing the dependent variables. Age, gender, HISS score, weekday of admission, alcohol consumption and BAC were included as covariates in the multivariate logistic regression models. In case of missing values we discarded the entire observation for the multivariate analyses. The resulting coefficients were exponentiated to obtain odds-ratios. Confidence intervals were calculated. A p-value less than 0.05 was considered statistically significant. R v 11.1 was used for statistical analyses [18].

## Results

### Patients

This study includes 860 adult patients with minimal, mild and moderate TBI, giving an estimated annual incidence of 179/100 000. The mean age was 40.9 years (range 15 - 99 years) and 66.6% were men. The mean age of men included in this study was significantly lower than the mean age of women (Mann-Whitney U test, p < 0.001). According to HISS, 12.8% (110/860) had a minimal TBI, 71.4% (614/860) had a mild TBI and 15.8% (136/860) had a moderate TBI. Table 1 shows a summary of the patient characteristics.

**Table 1:** Patient characteristics (n = 860)

		Overall N = 860	Gender		Test statistic
			Female	Male	
			N = 287	N = 573	
**Age (years)**	Median	35.0	45.0	32.0	F_1,858 _= 30.51, P < 0.001^2^

**Age group (years)**	15 - 24	292 (34.0%)	83 (28.9%)	209 (36.5%)	χ^2^_3 _= 52.89, P < 0.001^3^
	
	25 - 39	181 (21.0%)	42 (14.6%)	139 (24.3%)	
	
	40 - 59	205 (23.8%)	61 (21.3%)	144 (25.1%)	
	
	≥ 60	182 (21.2%)	101 (35.2%)	81 (14.1%)	

**HISS**^1^	Minimal	110 (12.8%)	54 (18.8%)	56 (9.8%)	χ^2^_2 _= 16.14, P < 0.001^3^
	
	Mild	614 (71.4%)	198 (69.0%)	416 (72.6%)	
	
	Moderate	136 (15.8%)	35 (12.2%)	101 (17.6%)	

**Alcohol consumption**	Yes	308 (35.8%)	62 (21.6%)	246 (42.9%)	χ^2^_1 _= 37.84, P < 0.001^3^
	
	No	552 (64.2%)	225 (78.4%)	327 (57.1%)	

^1 ^HISS - Head Injury Severity Scale [14]

^2 ^Wilcoxon test

^3 ^Pearson test

### Alcohol consumption

At time of admission, 35.8% (308/860) had consumed alcohol (Table 1). Using univariate and multivariate analysis we found that young age, male gender, trauma occurring during the weekends and mild and moderate TBIs were independent factors significantly associated with alcohol consumption (Table 2).

**Table 2:** Logistic regression analysis of variables possibly associated with increased probability of alcohol consumption

Variable	Univariate Odds ratio (95% CI)	Multivariate Odds ratio (95% CI)
**Age**	0.99*** (0.98,0.99)	0.98*** (0.97,0.99)

**Gender**		
Male	1	1
Female	0.46*** (0.32,0.66)	0.37*** (0.26,0.51)

**HISS**		
Minimal	1	1
Mild	4.84*** (2.47,9.51)	5.21*** (2.73,9.91)
Moderate	9.03*** (4.24,19.20)	10.13*** (5.00,20.55)

**Day**		
Monday	1	1
Tuesday	0.76 (0.38,1.54)	0.80 (0.41,1.57)
Wednesday	0.72 (0.37,1.42)	0.82 (0.43,1.55)
Thursday	0.71 (0.35,1.44)	0.76 (0.39,1.50)
Friday	0.94 (0.51,1.74)	0.92 (0.60,1.95)
Saturday	2.57*** (1.47,4.49)	3.18*** (1.87,5.40)
Sunday	3.24*** (1.86,5.67)	3.85*** (2.27,6.52)

*** P<0.001, ** P<0.01, * P<0.05

### Blood alcohol concentration

The blood alcohol concentration (BAC) was measured in 87% (267/308) of the patients which reported that they had consumed alcohol and/or were clinically judged by the admitting physician to be under the influence of alcohol. All 267 BACs measured were above 0, with a minimum value of 0.10‰ and a maximum value of 4.70‰. Of these, 7.9% (21/267) had a BAC in the range 0.10-0.99‰, 30.3% (81/267) had BAC 1.00-1.99‰ and 61.8% (165/267) had BAC ≥ 2.00‰. The mean BAC in the patients being under influence was 2.14‰. The mean BAC was 2.04‰ for women and 2.17‰ for men, which is not significantly different (independent samples t-test, p = 0.29).

### SNC guideline compliance

The overall compliance to the guidelines was 60.5% (520/860). Among the 340 patients not managed according to the guidelines, 88.2% (300/340) underwent over-triage and 11.8% (40/340) under-triage. In minimal, mild and moderate head injuries according to HISS the compliance were 45.5%, 54.9% and 97.8%, respectively. Using univariate and multivariate analysis we found that old age and moderate TBI were independent variables significantly associated with higher compliance rate (Table 3, Table 4). Neither gender nor alcohol influence nor BAC-level showed significant correlation with guideline compliance.

**Table 3:** Logistic regression analysis of variables possibly associated with increased probability of guideline compliance

Variable	Univariate Odds ratio (95% CI)	Multivariate Odds ratio (95% CI)
**Alcohol consumption**		
No	1	1
Yes	0.67* (0.49,0.93)	0.92 (0.69,1.22)

**Age**	1.01** (1.00,1.02)	1.01*** (1.01,1.02)

**Gender**		
Male	1	1
Female	0.84 (0.61,1.16)	0.87 (0.65,1.16)

**HISS**		
Minimal	1	1
Mild	1.61* (1.05,2.45)	1.46 (0.97,2.20)
Moderate	61.97*** (18.29,209.96)	53.20*** (15.96,177.38)

*** P<0.001, ** P<0.01, * P<0.05

**Table 4:** Logistic regression analysis of variables possibly associated with increased probability of guideline compliance in the 287 patients where BAC was measured

Variable	Univariate Odds ratio (95%CI)	Multivariate Odds ratio (95% CI)
**BAC**	0.93 (0.65,1.35)	1.30 (0.96,1.75)

**Age**	1.01 (1.00,1.03)	1.01*** (1.01,1.02)

**Gender**		
Male	1	1
Female	0.84 (0.42,1.66)	0.87 (0.65,1.16)

**HISS**		
Minimal	1	1
Mild	1.78 (0.42,7.54)	1.46 (0.97,2.20)
Moderate	146.04*** (12.64,1687.30)	53.20*** (15.96,177.38)

*** P<0.001, ** P<0.01, * P<0.05

## Discussion

This study confirms that alcohol consumption is common among patients with head injuries, and it shows that physician's guideline compliance is not affected by patients' alcohol consumption. Furthermore, we found that young age, male gender, trauma occurring during the weekends, mild and moderate TBIs were independent factors significantly associated with alcohol consumption.

The incidence of minimal, mild and moderate head injuries referred to hospital was estimated to 179/100 000, which is comparable to other studies reporting incidence levels in the range of 83.3 - 403/100 000 [3,5-8]. We report that the majority of the head injured patients were men (66.6%), and that most patients (34%) were aged 15-24. The majority of the patients, both those who had and had not consumed alcohol, were classified with a mild head injury. These findings are in line with the results from other studies of hospital referred head injuries [4,6-8,19].

We report that 35.8% of the patients had consumed alcohol at the time of admission. For traumas in general, it has been reported that 4 - 45% of injured patients are under the influence of alcohol [12,20,21]. With regards to traumatic brain injuries, alcohol use has been reported to be involved in 16 - 51% of these injuries [9-12].

Among the patients having consumed alcohol, 7.9% had a BAC lower than 1.00‰, 30.3% had a BAC between 1.00-1.90‰ and 61.8% a BAC at or greater than 2.00‰. The mean BAC was 2.14‰. Moskowitz reports that impairment in behavior, visual functions and body balance have been demonstrated at blood alcohol concentrations of 0.30-0.40‰ [22]. Increasing BAC aggravates these effects of alcohol [23]. Alcohol consumption resulting in intoxication might alter judgment, cause a more risk taking behavior, and impair motor and sensor functions, which can make people prone to head injuries.

Increasing levels of BACs can affect a person's memory [23] and cause amnesia, which could alter the classification of a head injury as defined in HISS [23,14]. There is a lower proportion of minimal head injury among the patients that had consumed alcohol than in those who had not. In the patient group being under the influence of alcohol, mild and moderate head injuries were more common. These findings might suggest that influenced patients can get lower GCS scores, and/or more often report loss of consciousness than the non-indulgent patients, which both will alter the HISS grade. However, firm evidence for reduction of GCS in trauma patients by alcohol is lacking. Thus, attributing low GCS to alcohol intoxication in TBI patients may delay necessary diagnostic and therapeutic interventions [24-26].

Guidelines are made in hope to secure safe, high quality and cost-effective patient management. Compliance to such guidelines is often low, as has been the case for the SNC-guidelines [15,16]. We report an overall guideline compliance of 60.5%, and that the main violation was over-triage. Alcohol consumption among the patients did not change the physicians' decision making with regards to guideline compliance. We found that the compliance rate was significantly higher for patients with moderate TBI than for patients with minimal or mild TBI, as has been shown by Heskestad et al. earlier [16].

This study has limitations. The study design was retrospective. The patients included were selected by general practitioners for hospital referral. The blood alcohol concentration was not measured in all relevant patients.

## Conclusions

This study confirms that alcohol consumption is common among patients with head injuries. Most of the patients who had consumed alcohol had blood alcohol concentrations at intoxication levels (BAC ≥ 1.00‰). The physician's guideline compliance was not affected by the patient's alcohol consumption. Alcohol consumption cannot explain the low guideline compliance.

## Competing interests

The authors declare that they have no competing interests.

## Authors' contributions

MEH was involved in the study design, gathered data, and drafted the manuscript. BH took part in the study design and gathering of data and helped draft the manuscript. TI participated in the study design and helped drafting the manuscript. BR participated in the study design and helped drafting the manuscript. PR did all statistical analysis and helped to draft the manuscript. EH contributed to the design of the study, data gathering and helped to draft the manuscript. All authors have read and approved the final manuscript.
